# Electrochemical Behavior of Saturated Potassium Nitrate Salts with Boron-Doped Diamond Electrode

**DOI:** 10.1021/acsaenm.5c00239

**Published:** 2025-06-27

**Authors:** Rene Pfeifer, Ondrej Szabo, Dhananjay K. Sharma, Johannes Eidenschink, Frank-Michael Matysik, Alexander Kromka

**Affiliations:** 1 86889 Institute of Physics, Czech Academy of Sciences, Cukrovarnicka 10, Prague 6 162 00, Czech Republic; 2 Faculty of Electrical Engineering, Czech Technical University in Prague, Technická 2, Prague 6 16200, Czech Republic; 3 Institute of Analytical Chemistry, Chemo- and Biosensors, 9147 University of Regensburg, Universitätsstraße 31, Regensburg 93053, Germany

**Keywords:** boron-doped diamond, electrochemistry, concentrated solar power tower, potassium nitrate, water-in-salt, electrochemical stability window

## Abstract

Potassium nitrate (KNO_3_) is widely utilized in concentrated solar power tower (CSPT) fuels and as an additive to address the issue of lithium dendrite formation in lithium-ion batteries and CSPT systems. This study investigates the influence of boron-doped diamond (BDD) films and their interaction with KNO_3_-saturated solutions to enhance these devices’ efficiency and energy capacity. One critical factor in achieving higher energy devices is the electrochemical stability window (ESW). Consequently, this research examines various properties of BDD, including grain size, doping level, conductivity, carbon-sp^3^/sp^2^ ratio, and electrolyte characteristics such as conductivity and pH. The results demonstrate that a BDD film grown at a B/C ratio of 2000 ppm, a grain size of 1.75 μm and a carbon-sp^3^/sp^2^ ratio value of 0.31 achieved the broadest ESW of 3.5 V. Advantageously, the properties of the KNO_3_ electrolyte, specifically concentration and pH, had minimal impact on the ESW. Controversially, the ionic conductivity of the electrolyte increased with concentration, with a peak value of 204 mS cm^–1^ observed in the supersaturated solution (3.78 mol kg^–1^).

## Graphical abstract

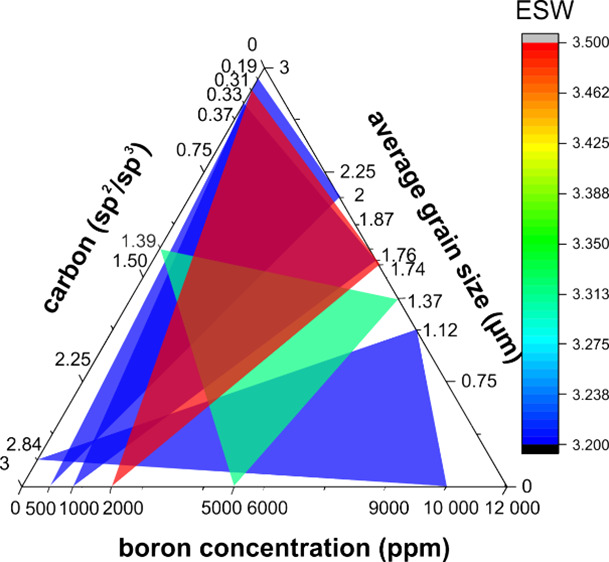

## 1 Introduction

KNO_3_ is a versatile chemical compound employed in a wide range of industries. This compound plays an essential role in the production of fertilizers, the manufacture of fireworks, the preservation of food, the production of gunpowder, the preparation of medicine and the formulation of toothpaste. It also serves as an oxidizer in amateur rocket propellants.^1^ Among alkali metal nitrates, KNO_3_ exhibits the highest melting point (334 °C) and lowest solubility (38.2 g per 100g of water at 25 °C).^2^ Due to their limited solubility, lithium and sodium nitrates are frequently preferred in water-in-salt (WiS) formulations for developing energy storage devices, including supercapacitors and batteries.^3–5^ These devices can achieve a maximum potential range between 2.56 and 3.0 V. Recently, KNO_3_ has been employed as an additive to mitigate the formation of lithium dendrites in lithium-battery systems.^6^

In industrial applications, a mixture of sulfuric acid (H_2_SO_4_) and KNO_3_ heated to 200 °C is a standard cleaning solution for removing sp^2^-carbon from diamond films.^7,8^ Additionally, molten KNO_3_ has been demonstrated as a viable medium for oxidative diamond etching.^9,10^

Furthermore, CSPT plants rely on working fluids containing a mixture of potassium and sodium nitrates (KNO_3_/NaNO_3_) for efficient energy generation.^11^ KNO_3_ is a preferred supporting electrolyte in electrochemistry, particularly for double-junction reference electrodes, especially when saturated potassium chloride (KCl) cannot be utilized.^12^ The diverse applications of KNO_3_ underscore its significance across a broad spectrum of scientific and industrial domains.

BDD electrodes have garnered significant interest in sensors, energy harvesting, and supercapacitors due to their low background current, a substantial ESW in aqueous (3–3.5 V) and non-aqueous media (5–7.5 V), resistance to corrosion and fouling and broad compatibility with environmental and biological settings.^13^

The primary methods for depositing BDD films onto various substrates involve hot-filament or microwave plasma-assisted chemical vapor depositions, which more or less deliver almost the same BDD properties.^13^ Here, the morphology of the film and its composition concerning non-diamond carbon (C-sp^2^) and boron-doping level play pivotal roles in determining the overall properties of the BDD.^13–15^

To enhance the quality of BDD films and precisely regulate the boron-density effects, the microwave-plasma-enhanced chemical vapor deposition (MECVD) method has proven to be highly effective. Consequently, boron substitutional doping in the diamond lattice via MECVD not only alters the electronic properties but also significantly influences the surface morphology and growth rate of the diamond films.^16–18^

Previous studies have revealed that high-quality (HQ) polycrystalline BDD with a lower B/C ratio (approximately 250 ppm) exhibits the broadest ESW within a doping range of 250 to 8000 ppm, utilizing a 0.5 M sulfuric acid (H_2_SO_4_) solution as the electrolyte.^19^

However, recent studies have surprisingly highlighted that low-doped polycrystalline BDD (containing 0.1% boron), which also contains elevated levels of carbon-sp^2^, demonstrates an exceptional ESW of 5.19 V on a 0.1 M H_2_SO_4_ electrolyte.^13^ This finding demonstrated that the presence of C-sp^2^ impurities is another crucial factor.

In recent research, Zelensky and co-workers investigated the surface activity of as-grown polycrystalline BDD using scanning electrochemical microscopy (SECM), revealing a heterogeneous distribution of surface activity, with specific spots exhibiting heightened electrochemical reactivity correlated with an increase in boron-doping levels.^20^ The researchers proposed utilizing chemical–mechanical (CM) polishing on BDD electrodes to achieve a more uniform distribution of the electrochemical surface activity. However, the impact of CM polishing on the electrochemical surface area (ECSA) remains unexplored.

The influence of the electrolyte on the ESW of BDD is a topic that received limited attention in the existing literature. Manzo-Robledo and co-workers have addressed this gap by examining the distinct behaviors of diluted solutions of potassium and sodium chlorides with a constant ionic strength.^21^ Their findings suggest that for the same anion and concentration, potassium tends to reduce the overpotential for oxygen evolution reactions (OERs) while increasing the overpotential for hydrogen evolution reactions (HERs). Additionally, increasing the concentration of KNO_3_ in a KCl solution tends to decrease the overpotential for HER due to the presence of nitrate (NO_3_^–^) anions in the solution. However, no study has been conducted to investigate the impact of a high range of KNO_3_ concentrations on the ESW of BDD electrodes.

This study aims to address a gap in the current literature by investigating the effects of KNO_3_ concentration, ranging from 0.5 to 3.8 mol kg^–1^, on the ESW of BDD electrodes. In addition, the study evaluates the relationship between the physical and chemical properties of microcrystalline BDD films—such as grain size, resistivity, boron-to-carbon (B/C) ratio, and sp^2^/sp^3^ ratio—and the properties of the KNO_3_ electrolyte, including pH and ionic conductivity. The goal is to determine the conditions that optimize the value of the ESW while minimizing the amount of electrolyte required to achieve it.

## 2 Experimental Section

### 2.1 Materials and Reagents

The ferrocene­(I) methanol (FcMeOH) used in the experiment was obtained from Sigma-Aldrich, Germany, and the KNO_3_ were sourced from Penta, Chrudim, Czech Republic. Both chemicals were of analytical grade and were used without any additional purification. Analytical-grade ethanol for UV analysis, acetone, and hydrochloric acid, all from Penta, were also used without further purification. Deionized water was used in all cases. A range of aqueous KNO_3_ solutions was prepared daily, with concentrations ranging from 0.5 to 3.78 mol kg^–1^. These solutions were stored in plastic vessels at an ambient temperature.

#### 2.1.1 Material Fabrication and Processing

All of the samples of unintentional doped diamond (UD) and BDD films were grown on silicon substrates (1 × 1 cm^2^). The substrates were thoroughly cleaned sequentially with acetone, isopropanol, and deionized water. The cleaning process was performed using an ultrasonic bath. Subsequently, the seeding step was carried out by immersing the substrates in a water-based nanodiamond powder suspension (NanoAmando), with an average particle size of 5 nm, using an ultrasound bath. The seeding process lasted for 40 min. Afterward, the substrates were rinsed again with deionized water and dried using a nitrogen-blowing technique.

### 2.2 Diamond Growth

In this study, both UD and BDD thin films were grown on commercially available p-type silicon substrates with a <100> orientation. The sample UD is unintentionally doped due to the boron contamination accumulated in the CVD chamber (memory effect) and no boron was used during the growth process.^22^ The growth process was carried out for 10 h using a clamshell MECVD system, specifically the SEKI SDSK6. As outlined in Table 1, the deposition conditions involved a power of 3 kW, total flow of the feed gases of 500 standard cubic centimeters per minute (sccm) and a gas pressure of 60 Torr along, with the Hall concentration and mobilities for all the UD and BDD thin films. The substrate temperature during the diamond film growth process was in the range of 970 to 1010 °C. For boron doping, trimethyl boron (TMB) was used as the dopant source, and the B/C ratio was varied between 500 and 10000 ppm, maintaining the same total flow (500 sccm).

**Table 1:** Parameters Used for the UD Diamond and BDD Depositions Using MECVD and B/C Ratio, Thickness (Determined by Scanning Electron Microscopy (SEM)), Resistivity, Hall Concentration, and Hall Mobility (Measured on As-Deposited Films)

sample	CH_4_/H_2_/TMB (in sccm)	B/C ratio (in ppm)	C/H ratio (in %)	thickness (μm)	sheet resistance (Ω/sq)	resistivity (Ω cm)	Hall Conc. (cm^–3^)	hall mobility cm^2^/(V s)
UD	10/490/0	0	0.98	5.7	5.40 × 10^4^	5.11	2.69 × 10^17^	4.55
BDD 1	10/487.5/2.5	500	0.99	6.2	2.30 × 10^3^	6.42 × 10^–1^	7.74 × 10^18^	1.26
BDD 2	10/485/5	1000	1.01	6.5	506	1.18 × 10^–1^	3.80 × 10^20^	0.14
BDD 3	10/480/10	2000	1.04	6.2	32.0	2.70 × 10^–2^	5.77 × 10^20^	0.40
BDD 4	10/465/25	5000	1.12	4.3	10.0	8.22 × 10^–3^	1.08 × 10^21^	0.70
BDD 5	10/440/50	10000	1.27	5.3	6.30	3.27 × 10^–3^	1.75 × 10^21^	1.10

### 2.3 Material Characterization

#### 2.3.1 SEM

All the as-deposited UD and BDD sample surfaces were morphologically characterized by field-emission SEM (FE-SEM) using MAIA3 TESCAN, operated at an accelerating voltage of 10 kV under a top-view configuration (0°) in the regime of secondary electrons. The thickness was evaluated by using the cross-sectional measurement of the fractured films and enabled the determination of the growth rate of thin films.

#### 2.3.2 Raman Spectroscopy

Raman spectra were obtained using an InViaReflex setup. The samples were excited by a continuous wave 432 nm laser with an intensity power of 2 mW. The laser was focused to a spot size of 10 μm on the sample perpendicular to the sample plane by using a 10× objective. The spectra were recorded for 10 s.

#### 2.3.3 Atomic Force Microscopy (AFM)

The morphology and roughness of the BDD films were evaluated by using AFM. The measurements were carried out on an Ntegra Prima AFM system (NTMDT) using high aspect ratio super sharp silicon tips covered with diamond-like carbon whiskers (NSG01_DLC, NTMDT). The AFM was operated at a frequency of 150 kHz with a nominal tip radius of 1–3 nm. The AFM images were acquired in a semicontact regime with a magnitude set point of 40%. The images had a resolution of 512 × 512 points, and the scan range was 20 × 20 μm^2^. The grain sizes of the particles and roughness were estimated by counting the detectable number of grains within a given area.

#### 2.3.4 Electrochemical Measurements

SECM measurements were carried out with an SECM 920C system from CH Instruments (Austin/TX, USA). The instrument was positioned on a dampening plate in a custom-made Faraday cage. The laboratory-constructed electrochemical cell was made from polytetrafluoroethylene. A three-electrode setup was applied, consisting of the SECM probe (as the working electrode), a Ag/AgCl 3 mol L^–1^ KCl reference electrode (CH Instruments, Austin/TX, USA), and a platinum wire as the counter electrode. Platinum disk electrodes with electrode diameters of 12.5 and an *R*_g_ value (defined as the ratio of the total tip radius and the radius of the active microdisk electrode) of >10 were used as SECM probes. BDD samples were mounted on the bottom of the electrochemical cell, which was leveled prior to the imaging experiments. Measurements were conducted in 5 mL of a mediator solution (1.5 mmol L^–1^ of ferrocene methanol, FcMeOH) with 1 mol L^–1^ KNO_3_ as a supporting electrolyte. Solutions were not deaerated before the measurements. Probe approach curves (PACs) were measured at a fixed probe potential corresponding to the respective mediator: +0.5 V FcMeOH. The maximum approach speed was 1 μm s^–1^, and the quiet time was 15 s. Imaging experiments were conducted with the same fixed probe potentials; the probe scan rate was 50 μm s^–1^, and the waiting time was 15 s. Areas covered in the images had a size of 500 × 500 μm^2^ with a step size of 5 μm and were recorded in constant-height mode, corresponding to feedback currents of either 50% (0 ppm substrate) or 150% (1000 up to 10000 ppm) relative to the steady-state current in the bulk solution. The image of the 500 ppm of substrate was recorded at a tip-to-surface distance of 3 μm.

Cyclic voltammetry (CV) measurements were carried out using a Metrohm AUTOLAB Potentiostat/Galvanostat PGSTAT302N with a frequency analyzer FRA32M module controlled by the software NOVA 2.1.4. Three-electrode cell configurations were used for all electrochemical measurements: working electrodes (BDD), counter-electrode (Pt), and reference electrode (No-leak Ag/AgCl—Model: 66-EE009, ESA Biosciences, Inc., USA). Before recording the data, each CV test was initially cycled five times to ensure a steady state of the system. The ESW was investigated for all KNO_3_ by CV, with a scan rate of 5 mV s^–1^ and a potential step of 10 mV. The current limit was set to ± 100 μA cm^–2^.

The BDD electrodes were fashioned employing a printed circuit board (copper film on Cuprextiti) as an electrode holder, a conductive paste consisting of Dotite Silver Paint D-550, and a polymer protective coating (ESL 240-SB) utilized for isolation. This method closely follows the procedures detailed in our prior works.^23^ All of the BDD electrodes had an electroactive area of 7 mm^2^.

Different concentrations of aqueous electrolytes of KNO_3_ were prepared for a range of 0.5 to 3.78 mol kg^–1^ of solution. The pH and conductivity of all electrolytes were measured by a pH meter (Denver Instrument 9342.1 UB-10, Goettingen, Germany, with pH electrode METTLER TOLEDO, LE422, Prague, Czech Republic) and Cond 3310 SET 1-Pocket conductometer, Model electrode: WTW TetraCon 325, Conductivity Cell (WTW, Xylem, Weilheim, Germany).

## 3 Results and Discussions

SEM analyses (Figure 1) reveal that all of the samples deposited by MECVD exhibit a microcrystalline nature with well-faceted crystallites. Additionally, the cross-sectional measurement (not shown) from the fractured surfaces of UD and BDD films confirms different film thicknesses, which is attributed to differences in the ratios of CH_4_/H_2_ and TMB during the deposition process. These observations align with previous studies demonstrating the influence of carbon content on the surface morphology and structure of microwave chemical vapor-deposited diamond thin films. In general, all the UD and BDD films show the microcrystalline nature of morphologies. However, the size of the grains tends to decrease with the increase in the carbon content (originating from TMB) from 500 to 10000 ppm amount of doping. Lloret et al. showed that more relaxed growth, i.e., bigger grain sizes, occurs at lower feed gas concentration.^24^ However, increasing the boron concentration (TMB content) in the amount of feed gas could change the growth conditions, which led these bigger grains to disappear, favoring an increase in the quantity of grains and also allowing the sharp edge lateral growth (as seen in BDD 4 and BDD 5). We observed that the grains got refined under highly boron-doping conditions as the feed gas mixture contains more diamond nucleation density due to boron doping. Moreover, the BDD 5 film exhibit too high boron concentration; thus, only a small portion of boron atoms can replace carbon atoms and enter the lattice of diamond films, while most other boron atoms accumulate at the crystal boundaries, which induce more structural defects, damage the integrity of grain, and affect the electric properties of the films. Besides, more activated boron atoms in a reactive atmosphere may hinder diamond growth on the substrate and reduce the percentage of diamond phase in the films.^25^ Using ImageJ, we calculated the average grain size of the UD and BDD films from the top-view surface acquired from SEM and are tabulated in Table 2. As mentioned earlier, we noticed film texture, the grains getting smaller in size, and enhancement of the grain boundaries with the addition of boron content in the feed gas. Moreover, we also observed the onset of graphitization as the TMB content increased (confirmed by Raman; see Figure 3). It is worth mentioning that BDD 4 and BDD 5 films were optically darker than other BDD films, and therefore, it is a reconfirmation of the graphitization (sp^2^) and concentration of boron at the abrupt grain boundaries, which increases light absorption.^26^ Furthermore, these findings show a direct correlation with the observation from the AFM studies (discussed in the next section). It is worth mentioning that the boron precursor also influences the growth rate, i.e., the growth rate will decrease compared to intrinsic diamond growth. Hence, we observed the abrupt film thickness from UD to BDD films (from cross-sectional) while increasing the doping level (500 ppm to 10000 ppm).^16^ We can conclude that it is crucial to use the appropriate amounts of boron doping concentration, which can influence the structural properties of BDD films and, consequently, their application for electrochemistry.

**Figure 1:**
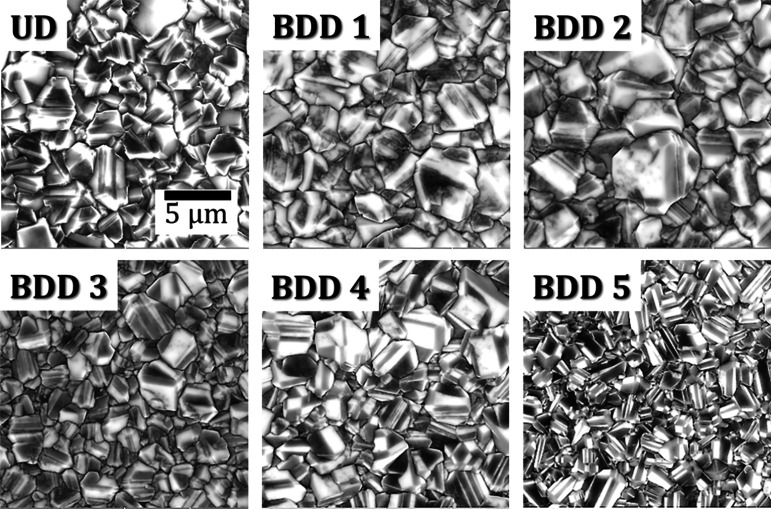
Comparison of surface morphologies via SEM micrographs of the UD and BDD 1 to BDD 5 films on silicon substrates. The scale for all the images is 5 μm.

**Table 2:** Diamond Peak Position, FWHM, Carbon-sp^2^/sp^3^ (from Raman) Content, and Average Grain Size Calculated from SEM and AFM^a^

samples	diamond peak position (cm^–1^)	fwhm of diamond peak	carbon-sp^2^/sp^3^ from Raman	residual stress (GPa)	grain size from SEM (μm)	grain size from AFM (μm)
***UD***	1330.25	6.89	0.3	0*	4.0	2.0
***BDD 1***	1329.35	6.73	0.2	1.6	3.0	2.1
***BDD 2***	1328.56	9.09	0.3	1.6	2.3	1.8
***BDD 3***	1328.90	7.60	0.3	1.6	1.9	1.7
***BDD 4***	1326.26	12.6	1.3	5.0	2.1	1.3
***BDD 5***	1321.09	16.0	2.9	10.8	1.3	1.2

a Considering UD as stress-free diamond

AFM is a powerful measurement tool that allows accurate and non-destructive measurements of the topography with very high resolution. We used the AFM surface analysis of all of the BDD films extensively to study the top surface morphologies. As expected, we noticed that all the samples from BDD 1 to BDD 5 (Figure 2) films yielded consistent roughness ranging from 0.144 to 0.241 nm. Furthermore, we also evaluated the grain size distribution over the area of 20 × 20 μm^2^ and listed it in Table 2. We avoided the UD film as it is not a point of interest due to its lower conductivity than other BDD films. Figure S1 (see Supporting Information) reveals a notable decrease in average grain size from 1.7 μm (BDD 1) to 1.2 μm (BDD 5) with increasing boron doping levels.

**Figure 2:**
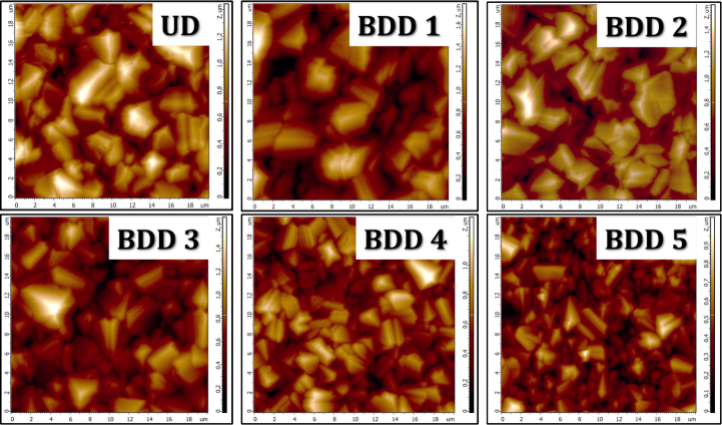
AFM images of the UD diamond and BDD 1 to BDD 5 films on silicon substrates.

Another crucial aspect in characterizing BDD films is evaluating the presence of non-diamond carbon and carbon-sp^3^ content within their structure. Raman measurements were conducted qualitatively to assess this parameter.

Raman measurements (Figure 3) were used to evaluate the diamond crystalline quality and the presence of non-diamond carbon phases. The characteristic sharp peak at approximately 1331.3 cm^–1^ represents the diamond signature in the UD sample. In the case of BDD samples, when the doping increased, the center of the characteristic peak (sp^3^–diamond, corresponding to the vibrations of two interpenetrating cubic sublattices) red shifts and becomes broader (increasing the FWHW). This indicates that the B atoms are well doped into the diamond lattice with the increase in the B doping content, resulting in a decrease in the sp^3^ content in the overall films (increasing carbon-sp^2^ vs sp^3^), as shown the Table 2. Meanwhile, we also observed the effect of boron doping on the diamond lattice with the increase in the peak observed at ∼500 cm^–1^(B_1_). However, the origin of this peak is unclear, but the positions roughly align with the two maxima in the phonon density of states (PDOS). However, we observed that peak B_1_ is a cumulative effect of two peak maxima along with some contribution of TPA. Literature suggests that this alignment may be linked to a relaxation of the wavevector selection rules, indicating that the peaks could be related to the actual incorporation of boron into the lattice rather than the hole concentration present in the overall film.^17,27,28^ It is worth mentioning that in UD film we observed a sharp peak ∼500 cm^–1^, which corresponds to the silicon peak of ∼521 cm^–1^ due to the background substrate arising from a longitudinal optical (LO) phonon. However, the intensity of the peak gradually decreased with increasing the B/C ratio (from 500 to 10000 ppm) as the feed gas mixture varied. Moreover, we observe peak B_1_ dominating the silicon peak in the BDD 4 and BDD 5 samples. According to the formulas documented in the literature, we calculate that there is an internal stress, as shown in Table 2, which is due to the lattice distortion caused by the boron atoms after entering the lattice of the diamond (considering UD as stress-free diamond films for our studies).^29^

**Figure 3:**
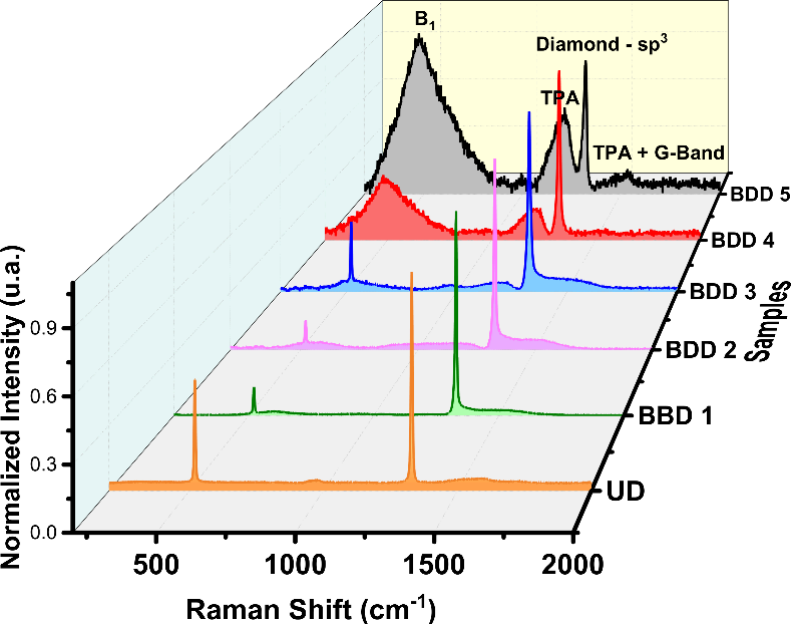
Raman spectra of UD and BDD samples showing a sharp diamond peak (sp^3^) at ∼1330 cm^–1^, and other bands are related to graphitic carbon (G-band) at ∼1580 cm^–1^ and TPA on grain boundaries.

Need not to mention, we also observed the presence of an amorphous sp^2^ (trans-polyacetylene (TPA) on the grain boundaries), graphite (G-band at ∼1540–1580 cm^–1^), and disordered graphite (D-band) in the samples.^30,31^ As expected, the UD film showed the highest quality of the defect-free diamond, which is consistent with literature findings.^32^

Finally, the Raman spectra of UD and BDD films obtained with different boron concentrations (B/C ratio) in the feed gas mixture clearly show boron incorporation in the films.

### 3.1 Electrochemical Measurements

SECM is a technique within the broader class of scanning probe microscopy (SPM) that is used to measure the local electrochemical behavior of liquid/solid, liquid/gas, and liquid/liquid interfaces. We performed SECM measurements to assess the electrochemical properties of the BDD films. Figure 4 displays the typical Probe Approach Curves (PACs) toward the as-grown BDD 1 electrode and a corresponding SECM image. During the approach toward the surface, positive, negative, and mixed feedbacks were observed. The red-dashed PAC represents positive feedback, indicating a conductive surface. Conversely, the blue-dashed PAC demonstrates a steady decrease in the current signal, which is characteristic of negative feedback from an insulating surface. Furthermore, Figure 4 illustrates the destinations of the PACs with red, blue, and green lines for the BDD 1 sample. The green line represents mixed feedback at the origin point (*x*_0_, *y*_0_). In contrast, negative feedback was observed for the coordinates (*x*_90_, *y*_45_), while positive feedback was noted for the coordinates (*x*_165_, *y*_180_).

**Figure 4:**
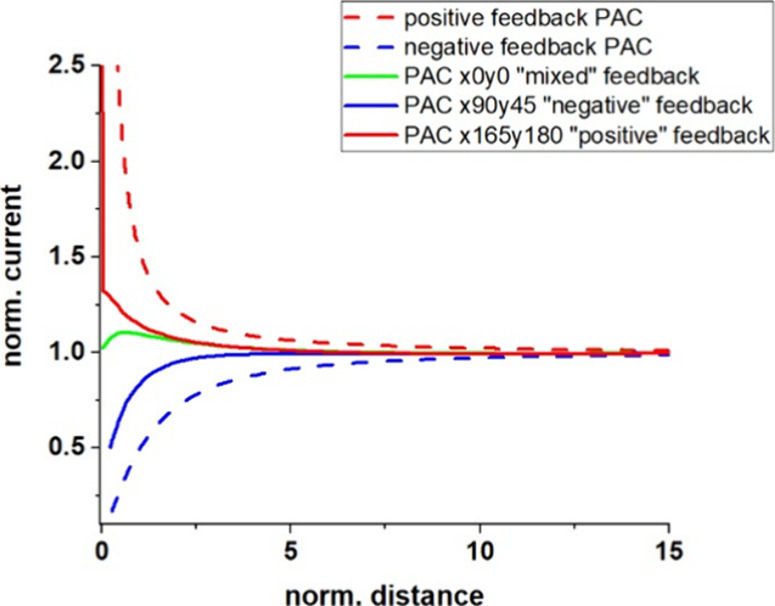
PACs towards the surface of the BBD 1 sample compared with the theoretical curves of positive and negative feedbacks. SECM probe was a Pt UME (*r* = 6.25 μm, *R*_g_ = 10). Probe potential was 0.5 V, quiet time was 15 s, and maximum approach speed was 1 μm s^–1^. The reference electrode was a Ag/AgCl (3 M KCl) electrode, and the counter-electrode was a Pt wire.

We conducted SECM measurements on all of the BDD samples to measure the electrochemical behavior locally at the grains/grain boundaries of the diamonds. In Figure 5, the SECM images visually represent the surfaces of as-grown UD to BDD 5 electrodes with a uniform normalized current scale. These images reveal heterogeneously distributed surface activity in the as-grown BDD films, characterized by regions of high electrochemical activity interspersed with spots that exhibit insulating properties, particularly at doping levels below 5000 ppm.

**Figure 5:**
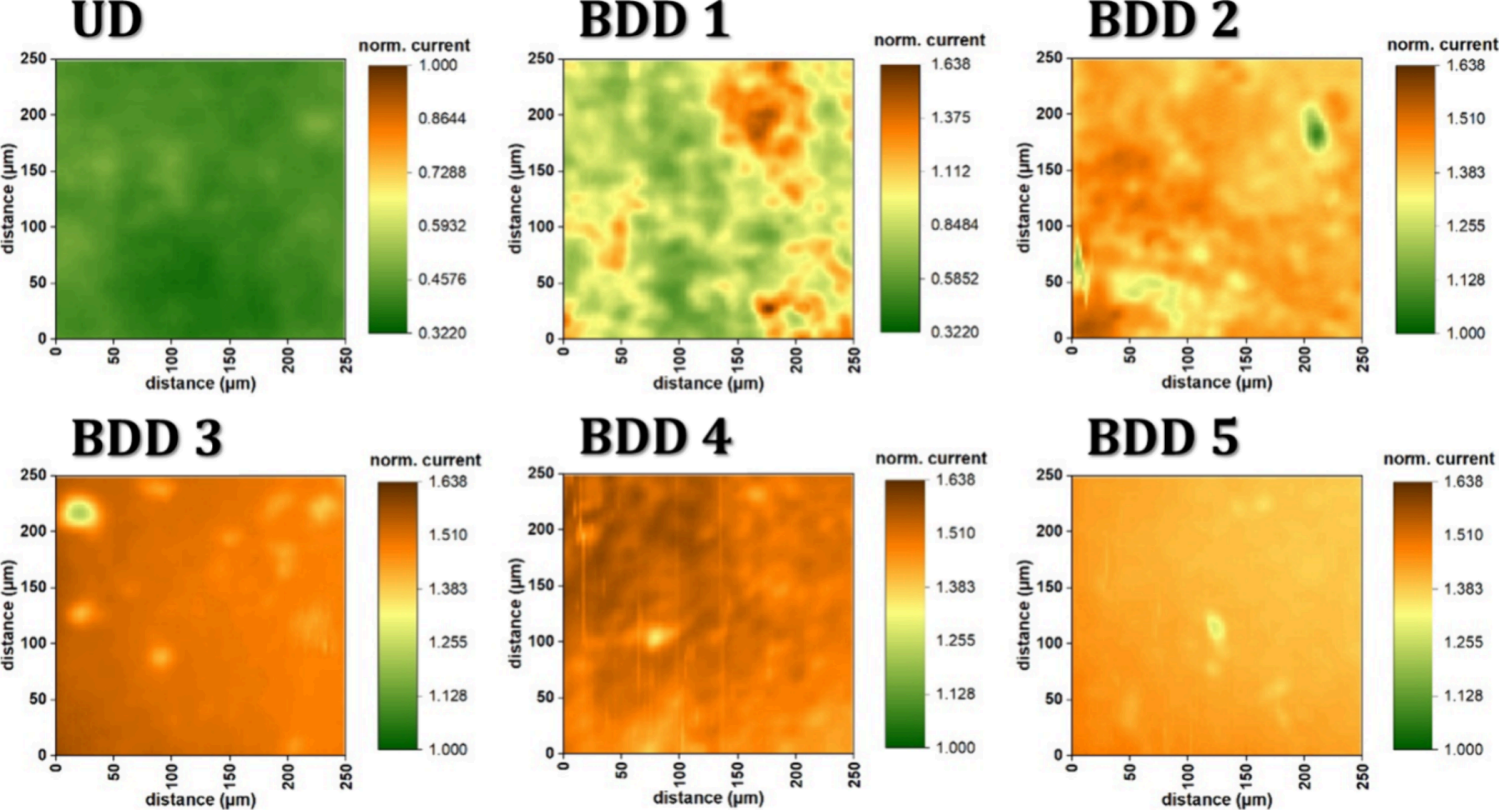
Feedback mode SECM images of BDD samples with different boron-doping levels ranging from 0 to 10,000 ppm. SECM probe was a Pt UME (*r* = 6.25 μm, *R*_g_ = 10). Probe potential was 0.5 V, quiet time was 15 s, and movement speed was 50 μm s^–1^. The starting height above the surface corresponds to the feedback current of 50% (UD) or 150% (BDD 2 to BDD5). The image of BDD 1 was recorded at a tip-to-surface distance of 3 μm. The reference electrode was a Ag/AgCl (3 M KCl) electrode, and the counter-electrode was a Pt wire.

It is worth noting that the UD film exhibits the lowest electrochemical activity, which aligns with expectations that this film is a highly UD diamond film, containing a very low amount of boron. In the case of BDD 5, characterized by the highest doping level with B/C 10000 ppm, it displays a homogeneous surface distribution. This finding is notable, as it was not previously reported in earlier studies.

These SECM images provide valuable insights into the electrochemical behavior and surface activity of the as-grown BDD films at various doping levels. The heterogeneous distribution of surface activity and the unique behavior observed in BDD 5 contribute to our understanding of the impact of doping on the electrochemical properties of the BDD films.

Furthermore, we established a relationship between the B/C ratio and the ESW in a supersaturated solution of 3.78 mol kg^–1^ of KNO_3_ (as shown in Figure 6). BDD 3 (B/C - 2000 ppm) demonstrated the highest ESW at 3.5 V. This finding diverges from observations made with H_2_SO_4_ as an electrolyte.^19^ This distinction can be attributed to BBD 3 acting as a transition point with increased surface activity, as observed through SECM, and a predominance of smaller diamond sizes, as observed via SEM (Figure 1).

**Figure 6:**
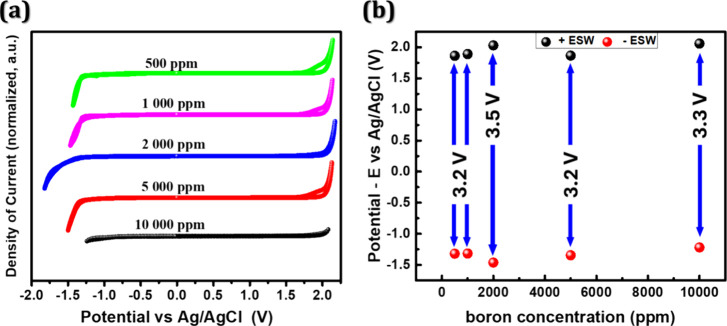
CVs (a) and ESW (b) for BDD 1 to BBD 5 electrodes for a concentration of 3.78 mol kg^–1^ of KNO_3_, recorded at 5 mV s^–1^.

The properties of the BDD film have a vital impact on the electrochemical properties. In this way, a correlation between resistivity, boron doping level, and ESW values is depicted in Figure 7. The smaller grain size corresponds to lower ESW values, and it can be concluded that the optimal boron-doping level is approximately B/C 2000 ppm.

**Figure 7:**
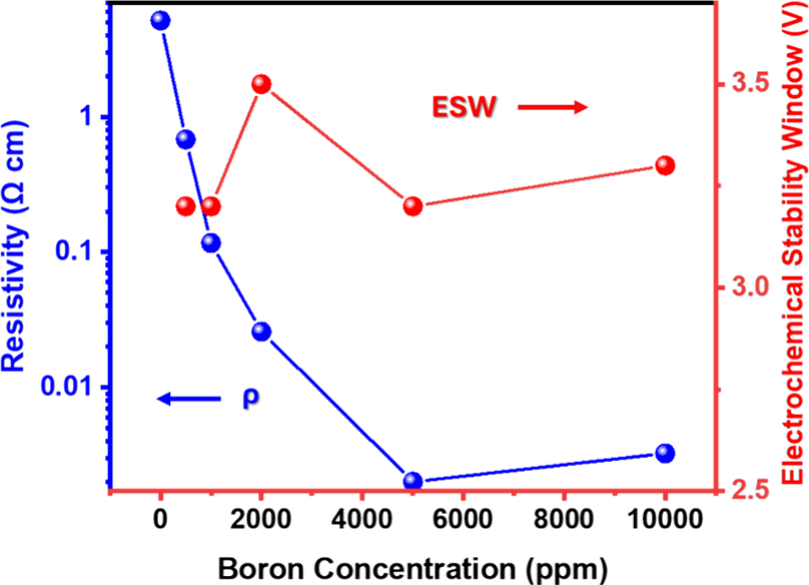
Resistivity and ESW dependence with boron concentration for all BDD films. The UD sample is removed as it is not a point of interest for these studies.

Based on these findings, BDD 3 was selected for subsequent experiments to evaluate the impact of the properties of the KNO_3_ electrolyte (concentration, pH, and conductivity) on the ESW.

The correlation between the ESW value and the concentration of KNO_3_ for BDD 3 is depicted in Figure 8. Notably, the ESW attains its peak value within the 2.0 to 3.78 mol kg^–1^ concentration range. The primary expansion of the ESW occurs in the region associated with HER. This observation aligns with the findings reported by Manzo-Robledo and co-workers.^21^ Nevertheless, no discernible changes are observed in the OER region.

**Figure 8:**
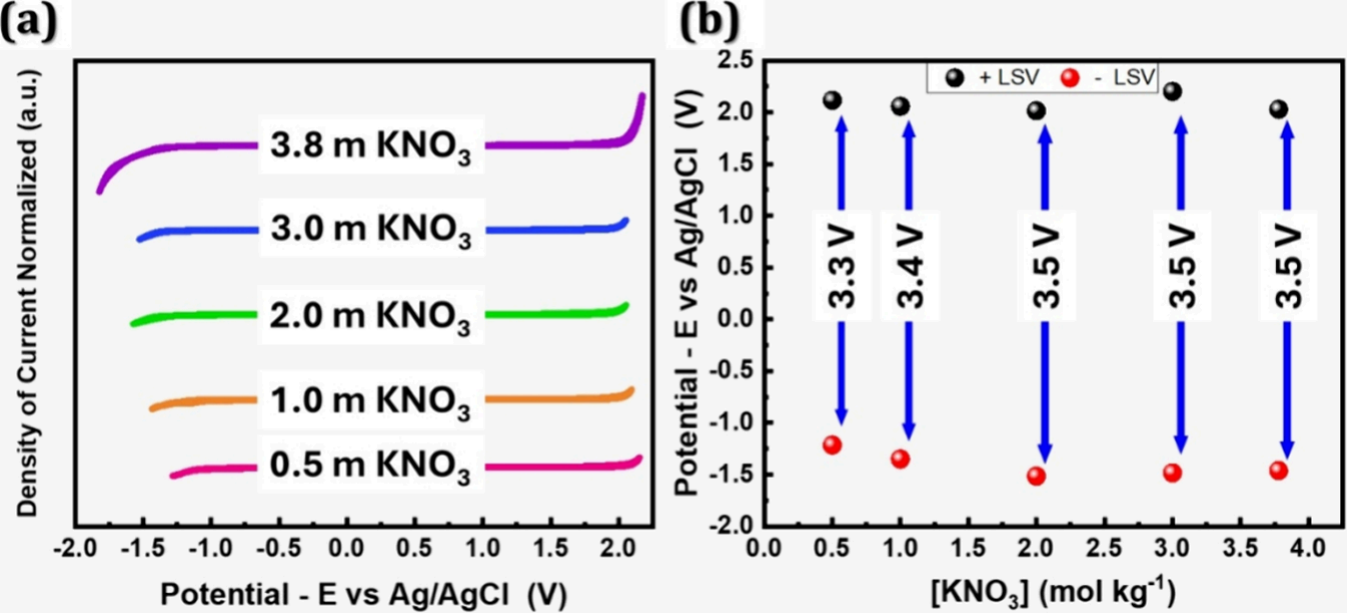
CVs (a,b) for different concentrations of KNO_3_ at a range of 0.5 to 3.8 mol kg^–1^, recorded at 5 mV s^–1^ for BDD3.

In the context of employing such a solution for power storage devices, a concentration of 2 mol kg^–1^ would be preferable, as it utilizes less material than the supersaturated solution. However, it is essential to consider other properties, such as pH and conductivity, as they may also play a significant role.

In this way, the dependence on pH and ionic conductivity was assessed and is depicted in Figure 9. It is evident that within this concentration range, the pH undergoes only slight variations, consistently maintaining a slightly acidic level. This fact broadens the use of this electrolyte on energy storage devices since it is less chemically aggressive to cathode and anode materials currently in use at this pH range. The ionic conductivity increases with concentration until it reaches its maximum value of 204 mS cm^–1^ for the supersaturated solution (3.78 mol kg^–1^). Different than for water-in-salt electrolytes (WiS), in which there is a drop of conductivity when the ESW increases, the KNO_3_ electrolyte achieves its maximum of ESW at a concentration of 2.0 mol kg^–1^, for a high conductivity value of 161 mS cm ^–1^. In this way, compared with prominent water-in-salt electrolytes for potassium–ion batteries, such as potassium acetate (KOAc) and KTFSI,^33^ KNO_3_ is cheaper and requires less material quantity to provide a slightly broader ESW (3.5 V). Moreover, this concentration could be also used as optimal quantity of KNO_3_ to be explored in solar power fuel cells, which currently rely on a quaternary mixture of NaNO_3_, KNO_3_, NaCl, and KCl to deliver its better efficiency rates.

**Figure 9:**
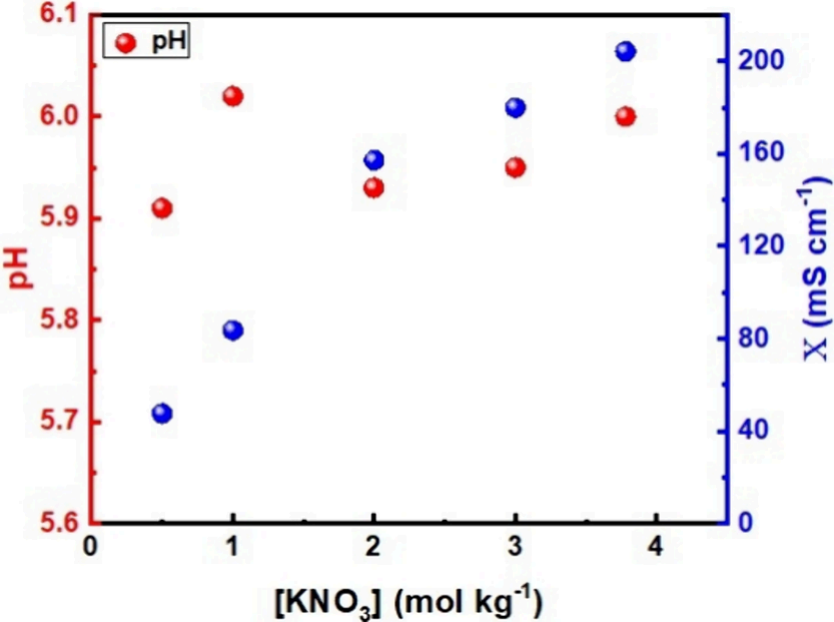
Dependence of pH and ionic conductivity (*X*) with the concentration of KNO_3_ in a range of 0.5 to 3.8 mol kg^–1^.

## 4 Conclusions

In this work, the relationship between the physical and chemical properties of microcrystalline BDD films—such as grain size, resistivity, boron-to-carbon (B/C) ratio, and sp^2^/sp^3^ ratio—and the properties of the KNO_3_ electrolyte, including pH and ionic conductivity, was evaluated. We observed that the grain sizes tend to decrease with the increase in the carbon content (originating from TMB) from 500 to 10000 ppm amount of doping. We found that Hall concentration for UD and heavy BDD 5 was 2.69 × 10^17^ and 1.7 × 10^21^, respectively. Furthermore, the Raman spectra show all the typical peaks associated with BDD films, and as expected, we noticed that the boron peak enhancing increases in the doping level. We extensively used SECM to reveal the heterogeneous surface activity across all as-grown BDD films, which clearly indicates the varying electrochemical properties within individual samples.

The investigation of ESW as a function of the KNO_3_ concentration yielded significant insights. BDD 3, characterized by a B/C ratio of 2000 ppm, exhibited the optimal ESW of 3.5 V, suggesting optimal doping level, also providing an ideal balance between conductivity and electrochemical stability. The ESWKNO_3_ concentration relationship reached a plateau at 3.5 V for 2.0 mol kg^–1^, indicating a limiting electrolyte concentration, beyond which no further ESW improvements were observed.

Electrolyte characterization revealed minimal pH variation (5.8–6.1) across the concentration range studied (0.5 to 3.8 mol kg^–1^ KNO_3_), while ionic conductivity demonstrated a positive correlation with concentration, peaking at 204 mS cm^–1^ for the supersaturated solution (3.78 mol kg^–1^). These findings underscore the importance of electrolyte properties in determining the electrochemical performance of BDD electrodes.

We conclude that the observed trends in grain size, boron doping levels, and electrochemical properties reported here for the first time provide valuable insights into the structure–property relationships in the BDD films. Our findings open vistas in designing and optimizing BDD electrodes for various electrochemical applications, particularly those requiring wide potential windows and stable performance in aqueous electrolytes.

## Supplementary Material



## Notes

Grain size distribution for UD Diamond and BDD1 to BBD5 films estimated by AFM (PDF)
