# Supplementary material for: Electrochemical Behavior of Saturated Potassium Nitrate Salts with Boron-Doped Diamond Electrode
Source: ACS Appl Eng Mater. 2025 Jun 27;3(7):2064–71. doi: 10.1021/acsaenm.5c00239 (PMC12305488; doi:10.1021/acsaenm.5c00239)
Supplement: Supplementary file 1 [file em5c00239_si_001.pdf]

Supporting Information:

Electrochemical behavior of Saturated Potassium Nitrate salts with boron-doped diamond electrode

Rene Pfeifer[1*], Ondrej Szabo[1], Dhananjay K. Sharma[1,2], Johannes Eidenschink[3], Frank-Michael Matysik[3], Alexander Kromka[1]

[1] Institute of Physics, Czech Academy of Sciences, Cukrovarnicka 10, 162 00, Prague 6, Czech Republic

[2] Faculty of Electrical Engineering, Czech Technical University in Prague, Technická 2, Prague 6, Czech Republic

[3] Institute of Analytical Chemistry, Chemo- and Biosensors, University of Regensburg, Universitätsstraße 31, 93053 Regensburg, Germany

*rpfeifer@fzu.cz

Contents	Page. No.
Grain size distribution for ID and BDD 1 to BBD 5 by AFM.	S1

Atomic Force Microscopy (AFM)

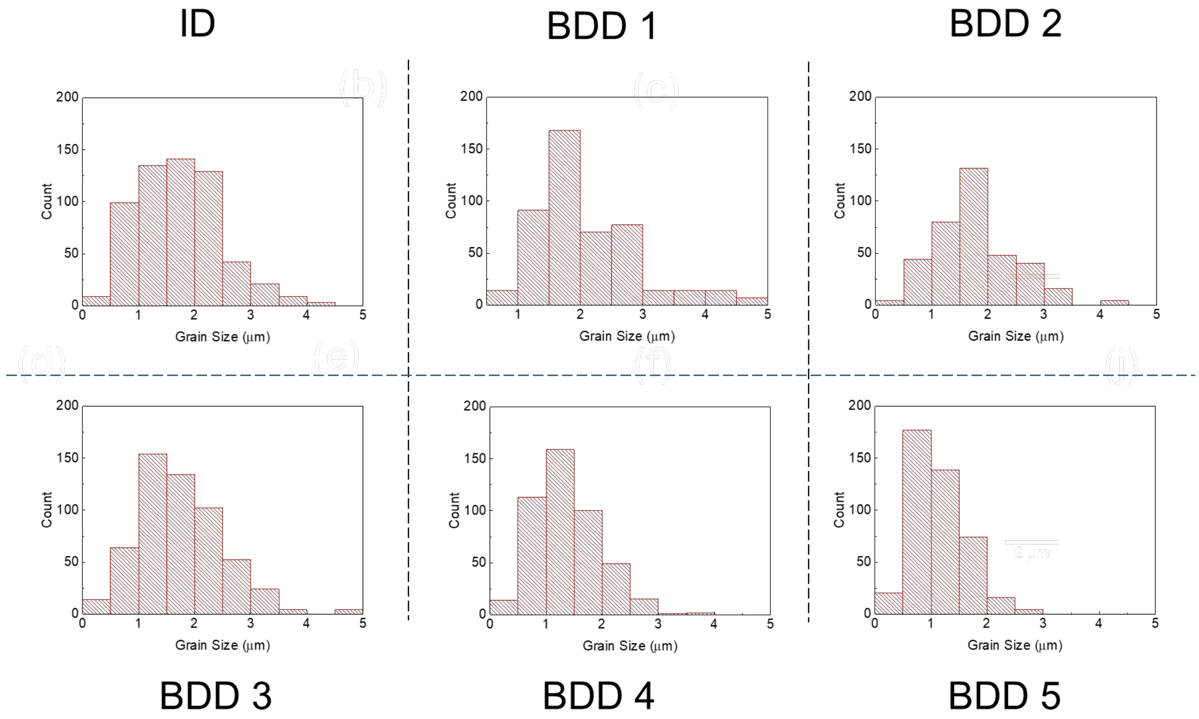

Figure S1: Grain size distribution for ID and BDD 1 to BBD 5 by AFM.
